# Comparative diagnostic accuracy of next‐generation sequencing in different specimen types for periprosthetic joint infection: A systematic review and meta‐analysis

**DOI:** 10.1002/ksa.70095

**Published:** 2025-10-17

**Authors:** Lina Wang, Shangxiang Feng, Zhongyuan Zhao, Yuchi Zhao, Shengjie Dong, Li Cao, Kun Song

**Affiliations:** ^1^ Department of Infectious Diseases Affiliated Hospital of Qingdao University Qingdao Shandong China; ^2^ Department of Joint Surgery Yantai City Yantaishan Hospital Yantai Shandong China; ^3^ Department of Orthopaedics The First Affiliated Hospital of Xinjiang Medical University Urumqi Xinjiang China

**Keywords:** diagnostic accuracy, meta‐analysis, next‐generation sequencing, periprosthetic joint infection, specimen type

## Abstract

**Purpose:**

Discrepancies in next‐generation sequencing (NGS) results across synovial fluid, periprosthetic tissues and implant sonicate fluid pose a significant clinical challenge in diagnosing periprosthetic joint infection (PJI). We conducted a systematic review and meta‐analysis to compare the diagnostic accuracy of these NGS sample types for PJI.

**Methods:**

This study adhered to the Preferred Reporting Items for Systematic Reviews and Meta‐Analyses guidelines. A comprehensive literature search was conducted in PubMed, EMBASE and the Cochrane Library databases from inception to 1 June 2025. Two independent reviewers performed data extraction and assessed study quality using the Quality Assessment of Diagnostic Accuracy Studies‐2 tool. For each specimen type, we estimated the pooled sensitivity and specificity, summary receiver operating characteristic (SROC) curve and the area under the SROC curve (AUC).

**Results:**

Following screening, 18 studies were included. Pooled sensitivities of NGS for PJI diagnosis were as follows: synovial fluid 0.86 (95% confidence interval [CI]: 0.79–0.91), periprosthetic tissue 0.86 (95% CI: 0.69–0.95) and sonicate fluid 0.89 (95% CI: 0.77–0.95). Corresponding specificities were 0.94 (95% CI: 0.91–0.96), 0.98 (95% CI: 0.85–1.00) and 0.96 (95% CI: 0.91–0.98). AUCs were 0.93 (0.89–0.95), 0.96 (0.88–0.97) and 0.96 (0.88–0.97), respectively. Pairwise comparisons of AUCs showed no statistically significant differences (*p* > 0.05).

**Conclusions:**

This meta‐analysis suggests that NGS of sonicate fluid demonstrates higher sensitivity compared to NGS of synovial fluid or periprosthetic tissue, while maintaining strong specificity, making it valuable for detecting infection. Periprosthetic tissue NGS demonstrated exceptional specificity and acceptable sensitivity, making it valuable for confirming infection. All specimens show clinically useful AUC values. Sonicate fluid shows promise, but specimen selection warrants careful consideration of the sensitivity‐specificity trade‐off in clinical practice and requires validation of clinical utility due to the absence of a perfect PJI diagnostic gold standard and the risk of false positives.

**Level of Evidence:**

Level II.

## Graphical abstract

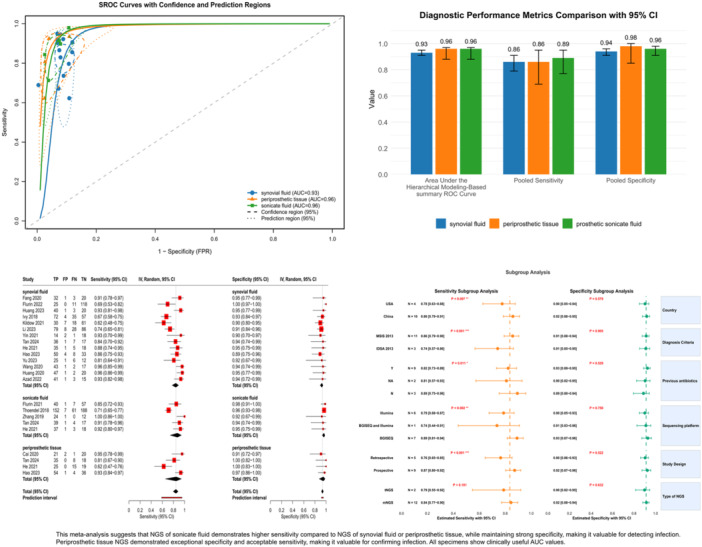

**Abbreviations**
AUC: area under the SROC curve
CI: confidence interval
IDSA: Infectious Diseases Society of America
mNGS: metagenomic next‐generation sequencing
MSIS: Musculoskeletal Infection Society Infectious
NGS: next‐generation sequencing
PJI: periprosthetic joint infection
PRISMA: Reporting Items for Systematic Reviews and Meta‐Analyses
PROSPERO: International Prospective Register of Systematic Reviews
QUADAS: Quality Assessment of Diagnostic Accuracy Studies
SROC: summary receiver‐operating characteristic
tNGS: targeted next‐generation sequencing

## INTRODUCTION

Periprosthetic joint infection (PJI) represents a devastating complication following joint arthroplasty, with reported incidence rates of 0.8%–1.9% after knee arthroplasty and 0.3%–1.7% after hip arthroplasty [8, 17]. Rapid and accurate pathogen identification is critical for guiding appropriate surgical interventions and selecting targeted antimicrobial therapy [18, 51]. Although conventional microbial culture remains the gold standard for PJI diagnosis, its limitations are evident in the high proportion (7%–42.1%) of culture‐negative PJI cases encountered in clinical practice [2, 6, 25, 26, 48]. Next‐generation sequencing (NGS), particularly metagenomic NGS (mNGS), has rapidly evolved into an extensively adopted and increasingly favoured technology for clinical pathogen detection [4]. This approach provides the theoretical capability to identify all microorganisms present in clinical specimens [7, 10, 40], with accumulating evidence demonstrating superior sensitivity and diagnostic accuracy compared to conventional culture methods [15, 42].

Although previous studies have reported variations in diagnostic accuracy between specimen types for culture‐based PJI diagnosis [47], the impact of sample type on NGS performance remains underexplored. Synovial fluid, periprosthetic tissues and sonicate fluid are the most commonly used specimen types for NGS‐based bacterial detection in PJI [17, 43]. Optimal specimen selection is critical for pathogen identification, applying equally to both conventional culture and molecular diagnostic techniques [9, 21, 27, 38]. While some studies suggest implant sonicate fluid improves diagnostic sensitivity [17, 43], consensus remains unclear regarding specimen‐dependent variations in NGS diagnostic performance. Therefore, understanding these differences is essential for accurate PJI diagnosis and optimal clinical management.

The primary clinical question of this systematic review was ‘Do significant differences exist in the diagnostic accuracy of NGS across specimen types for PJI diagnosis?’ We hypothesised that pooled diagnostic performance of NGS differs significantly among these specimen types. To address this, we conducted a systematic review and meta‐analysis comparing the diagnostic performance of NGS using synovial fluid, periprosthetic tissues and sonicate fluid specimens, quantifying pooled diagnostic accuracy differences between these sample types.

## MATERIALS AND METHODS

This systematic review and meta‐analysis adhered to the Preferred Reporting Items for Systematic Reviews and Meta‐Analyses (PRISMA) guidelines. The study protocol was prospectively registered with the International Prospective Register of Systematic Reviews (PROSPERO; registration number: CRD420251079770). Institutional review board approval was not required as this study synthesises existing published data.

### Search strategy

A comprehensive literature search was conducted in PubMed, EMBASE and the Cochrane Library databases from inception through 1 June 2025. The search strategy employed Boolean operators to combine the following conceptual domains: (1) PJI concepts: (‘prosthesis‐related infection’ OR ‘periprosthetic joint infection’ OR ‘prosthetic joint infection’ OR ‘PJI’ OR ‘prosthesis infection’ OR ‘septic loosening’ OR ‘periprosthetic infection’); (2) Next‐generation sequencing concepts: (‘high‐throughput nucleotide sequencing’ OR ‘next‐generation sequencing’ OR ‘NGS’ OR ‘metagenomic sequencing’ OR ‘metagenomic next‐generation sequencing’ OR ‘mNGS’ OR ‘shotgun metagenomics’ OR ‘targeted next‐generation sequencing’ OR ‘tNGS’); The final search syntax combined these domains as (#1) AND (#2). No language restrictions were applied. Complete database‐specific search strategies are detailed in Supporting Information: Material.

### Study screening and eligibility assessment

The inclusion criteria were as follows: (1) original diagnostic accuracy studies reporting sensitivity and specificity of NGS for PJI diagnosis using synovial fluid, periprosthetic tissues, or sonicate fluid; (2) diagnosis was based on internationally recognised criteria from the Musculoskeletal Infection Society (MSIS) [35], Infectious Diseases Society of America (IDSA) [33], International Consensus Meeting (ICM) [34] or European Bone and Joint Infection Society (EBJIS) [31] (Supporting Information: Material); and (3) sufficient data to reconstruct 2 × 2 contingency tables (TP, FP, FN and TN). Exclusion criteria encompassed non‐primary research (technical notes, editorials, commentaries, reviews and meta‐analyses), case reports (<5 cases), animal/in vitro studies and articles with inaccessible full texts. Two reviewers independently performed dual‐phase screening using Covidence® software: initial title/abstract screening against eligibility criteria, followed by full‐text assessment of potentially eligible manuscripts. Duplicates were removed via EndNote® with manual verification, and discrepancies were resolved through consensus discussion or third‐reviewer adjudication when required.

### Data extraction

Data extraction was independently performed by two reviewers using a piloted form, encompassing: authors, year of publication, country, study design, number of patients, diagnostic criteria, sequencing platform, type of NGS, type of arthroplasty, sample type and antibiotic exposure prior to sampling. Furthermore, for studies analysing multiple specimen types within the same cohort, each specimen dataset was extracted and analysed separately, provided outcomes were distinctly reported without patient overlap. Discrepancies in extraction were resolved through consensus or third‐reviewer adjudication.

### Data analyses

For each included study, a 2 × 2 contingency table was constructed detailing true positives (TP), true negatives (TN), false positives (FP) and false negatives (FN). Sensitivity and specificity estimates were summarised using forest plots. We performed bivariate random‐effects meta‐analysis using the Reitsma model [37] to generate summary receiver operating characteristic (SROC) curves, calculating the area under the curve (AUC) with 95% confidence intervals (CIs). Results were visualised on the SROC plane with 95% confidence region ellipses and 95% prediction region ellipses. Moreover, sensitivity analysis was performed to investigate potential sources of heterogeneity, and subgroup analysis was conducted to derive more specific conclusions. Publication bias was evaluated using funnel plots, with asymmetry indicating potential bias. A value of *p *< 0.05 was considered significant. All statistical analyses were conducted using Stata Statistical Software (StataCorp, release 16.0; StataCorp LP., College Station, TX), R (version 4.5.0), and Review Manager 5 (Cochrane, London, UK, version 5.4.0).

### Evaluation of bias risk

Two reviewers independently assessed study quality using the Quality Assessment of Diagnostic Accuracy Studies‐2 (QUADAS‐2) tool [49]. This instrument evaluates four domains: (1) patient selection, (2) index test, (3) reference standard and (4) flow and timing. Risk of bias and applicability concerns were rated as ‘low’, ‘high’ or ‘unclear’ for each domain. Disagreements were resolved through consensus‐based discussion. Publication bias was evaluated using funnel plots for diagnostic accuracy measures, supplemented by Deeks' asymmetry test.

## RESULTS

### Literature screening and identification

**Figure 1:**
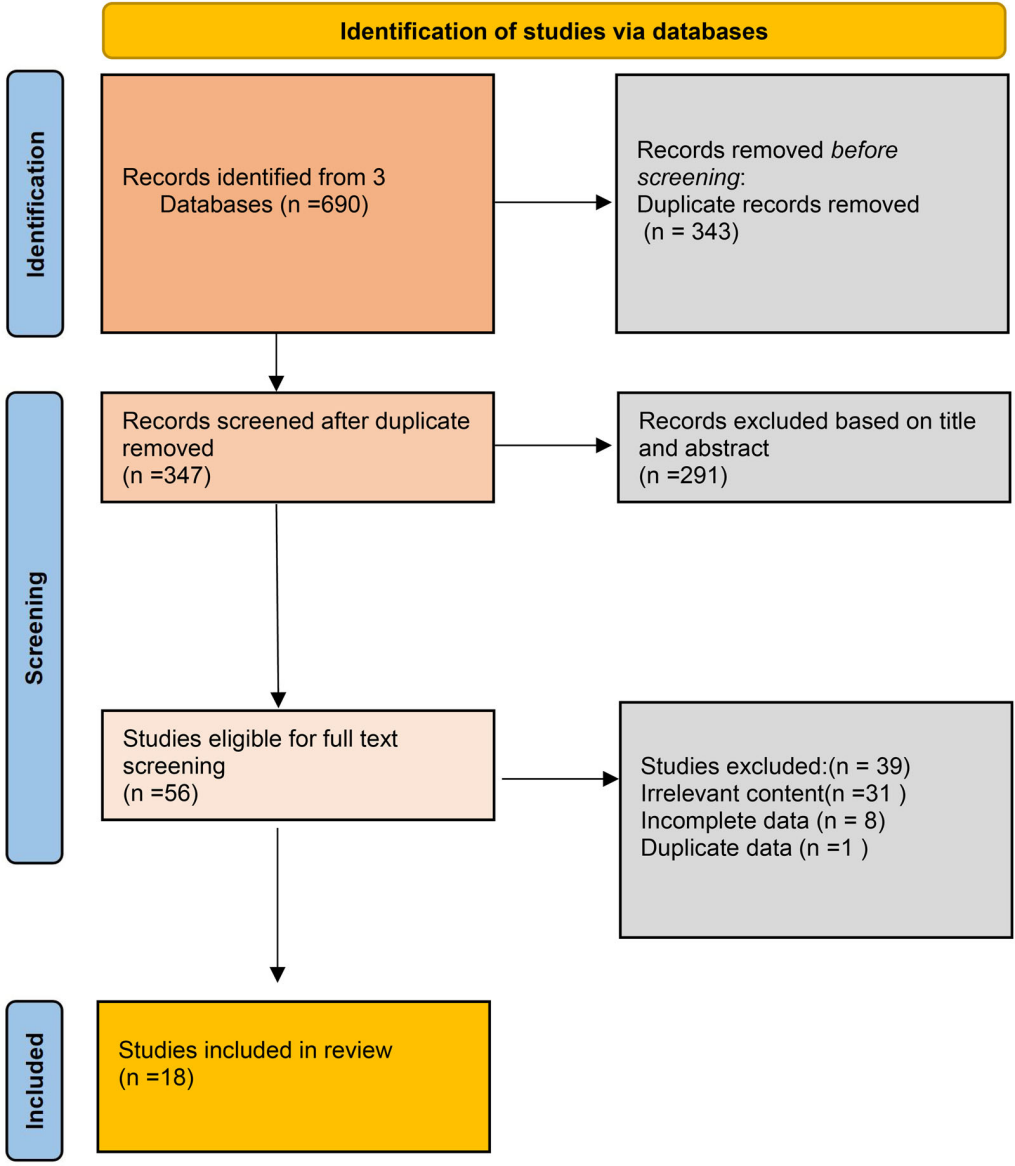
Preferred Reporting Items for Systemic Meta‐Analyses (PRISMA) flowchart for the study identification process.

Figure 1 presents the PRISMA flowchart detailing study selection. From an initial yield of 690 records identified through database searching, 343 duplicates were removed using EndNote®. After screening titles/abstracts of 347 unique records against eligibility criteria in Covidence®, 56 studies underwent full‐text assessment. Eighteen studies met all inclusion criteria and were included in the meta‐analysis.

### Study characteristics

Table 1 summarises the characteristics of included studies. Synovial fluid was analysed in 14 studies [1, 11, 12, 16, 17, 19, 20, 22, 24, 28, 43, 45, 50, 52], periprosthetic tissues in four studies [3, 16, 17, 43], and sonicate fluid in five studies [13, 17, 43, 44, 54]. Complete study characteristics stratified by specimen type are detailed in Supporting Information: Tables 1–3 and Figure S1.

**Table 1:** Characteristics of included studies.

Study (author & year)	Inclusion interval	Study design	Infected/control joints	Diagnosis criteria	Country	Sequencing platform	Type of NGS	Previous antibiotics	Sample type	Type of arthroplasty
Ivy et al. [22], 2018	04/1998–06/2017	Retrospective	107/61	IDSA 2013	USA	Illumina	mNGS	Y	SNV fluid	Knee: 168
Thoendel et al. [30], 2018	2011–2016	Retrospective	213/195	IDSA 2013	USA	Illumina	mNGS	Y	SON fluid	Knee: 281 Hip: 127
Zhang et al. [54], 2019	12/2016–12/2018	Prospective	24/13	MSIS 2013	China	BGISEQ	mNGS	Y	SON fluid	Knee: 19 Hip: 18
Cai et al. [3], 2020	07/2017–07/2019	Prospective	22/22	MSIS 2013	China	BGISEQ	mNGS	Y	PPT	Knee: 13 Hip: 31
Wang et al. [45], 2020	03/2017–07/2018	Prospective	45/18	MSIS 2013	China	BGISEQ	mNGS	N	SNV fluid	knee and Hip:NA
Fang et al. [11], 2020	06/2016–12/2018	Prospective	25/13	MSIS 2013	China	BGISEQ	mNGS	Y	SNV fluid	Knee: 19 Hip: 19
Huang et al. [20], 2020	03/2017–07/2018	Prospective	49/21	MSIS 2013	China	BGISEQ	mNGS	Y	SNV fluid	Knee: 36 Hip: 34
Kildow et al. [24], 2021	01/2017–07/2019	Retrospective	48/68	MSIS 2013	USA	Illumina	mNGS	N	SNV fluid	Knee: 92 Hip: 24
Flurin et al. [13], 2021	05/2007–09/2019	Retrospective	47/58	IDSA 2013	USA	Illumina	tNGS	Y	SON fluid	elbow: 105
He et al. [17], 2021	10/2017–04/2019	Prospective	40/19	MSIS 2013	China	BGISEQ	mNGS	Y	PPT, SNV fluid, SON fluid	Knee: 34 Hip: 25
Yin et al. [50], 2021	07/2017–12/2019	Prospective	15/20	MSIS 2013	China	BGISEQ	mNGS	NA	SNV fluid	Knee: 16 Hip: 19
Flurin et al. [12], 2022	08/2020–05/2021	Retrospective	36/118	IDSA 2013	USA	Illumina	tNGS	Y	SNV fluid	Knee: 96 Hip: 43 shoulder:14 elbow:1
Azad et al. [1], 2022	12/1998–06/2021	Retrospective	44/16	IDSA 2013	USA	Illumina	tNGS	NA	SNV fluid	Knee: 59
Huang et al. [19], 2023	04/2020– 09/2022	Retrospective	43/21	MSIS 2013	China	BGISEQ and Illumina	mNGS	Y	SNV fluid	Knee: 30 Hip: 34
Li et al. [28], 2023	04/2019–04/2021	Prospective	107/94	MSIS 2013	China	Illumina	mNGS	Y	SNV fluid	Knee: 140 Hip: 61
Yu et al. [52], 2023	05/2020–03/2022	Prospective	31/13	MSIS 2013	China	BGISEQ	mNGS	Y	SNV fluid	Knee: 36 Hip: 8
Hao et al. [16], 2023	01/2018–01/2021	Prospective	58/37	MSIS 2013	China	BGISEQ	mNGS	N	PPT, SNV fluid	Knee: 56 Hip: 39
Tan et al. [43], 2024	09/2021–09/2022	Prospective	43/18	MSIS 2013	China	Illumina	mNGS	Y	PPT, SNV fluid, SON fluid	Knee: 37 Hip: 24

Abbreviations: IDSA, Infectious Diseases Society of America; mNGS, metagenomic next‐generation sequencing; MSIS, Musculoskeletal Infection Society; N, no; NA, not available; PPT, periprosthetic tissues; SNV fluid, synovial fluid; SON fluid, sonicate fluid; tNGS, targeted next‐generation sequencing; Y, yes.

### Quality assessment

**Figure 2:**
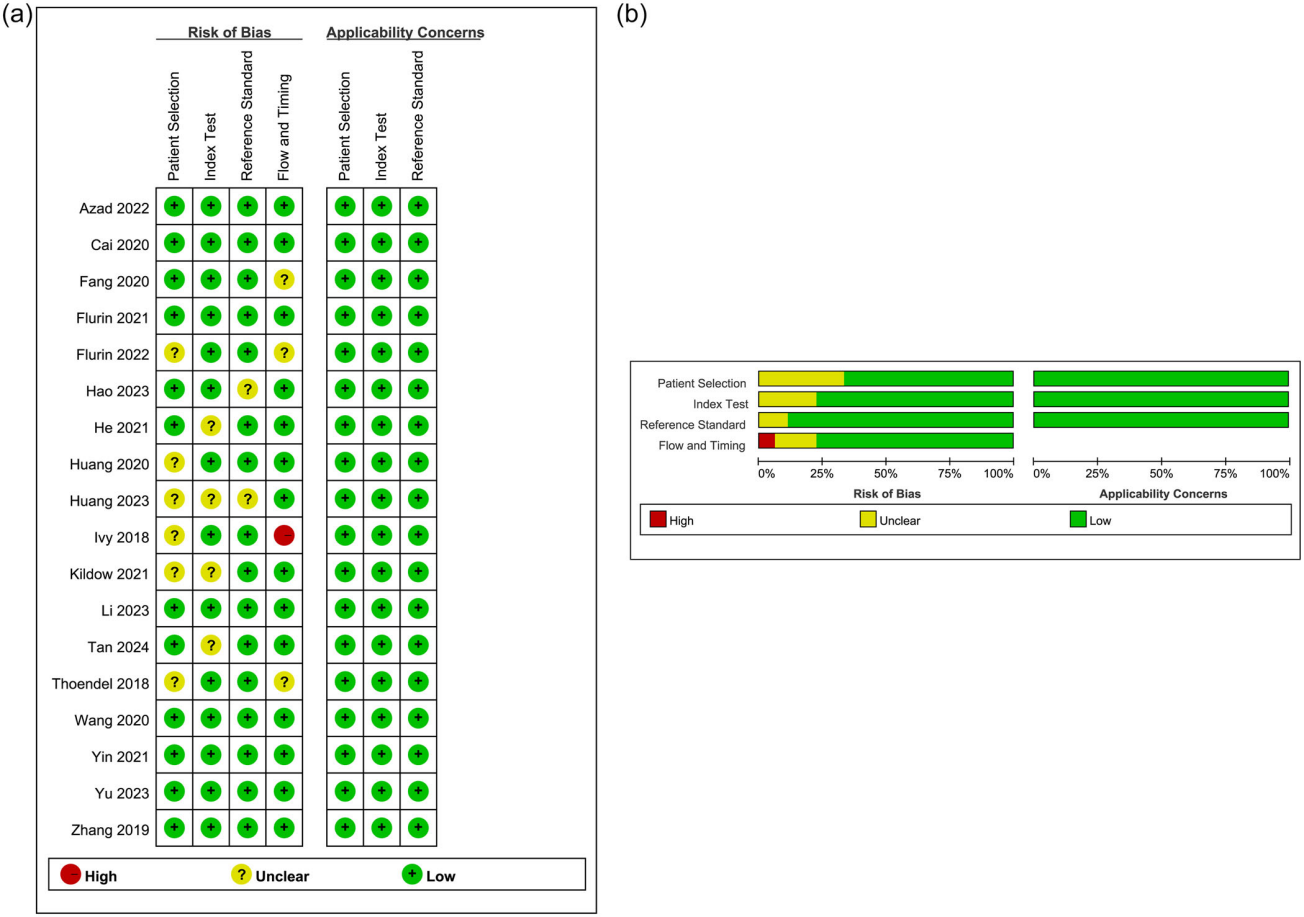
(a) Risk of bias and applicability concerns summary. (b) Risk of bias and applicability concerns graph.

The included studies demonstrated predominantly low risk of bias across three QUADAS‐2 domains: patient selection, index test (NGS methodology) and reference standard (PJI diagnostic criteria). A single study exhibited high risk of bias in flow and timing [32]. Applicability concerns were consistently low for all domains. These quality assessment results are comprehensively presented in Figure 2.

### Diagnostic performance

**Figure 3:**
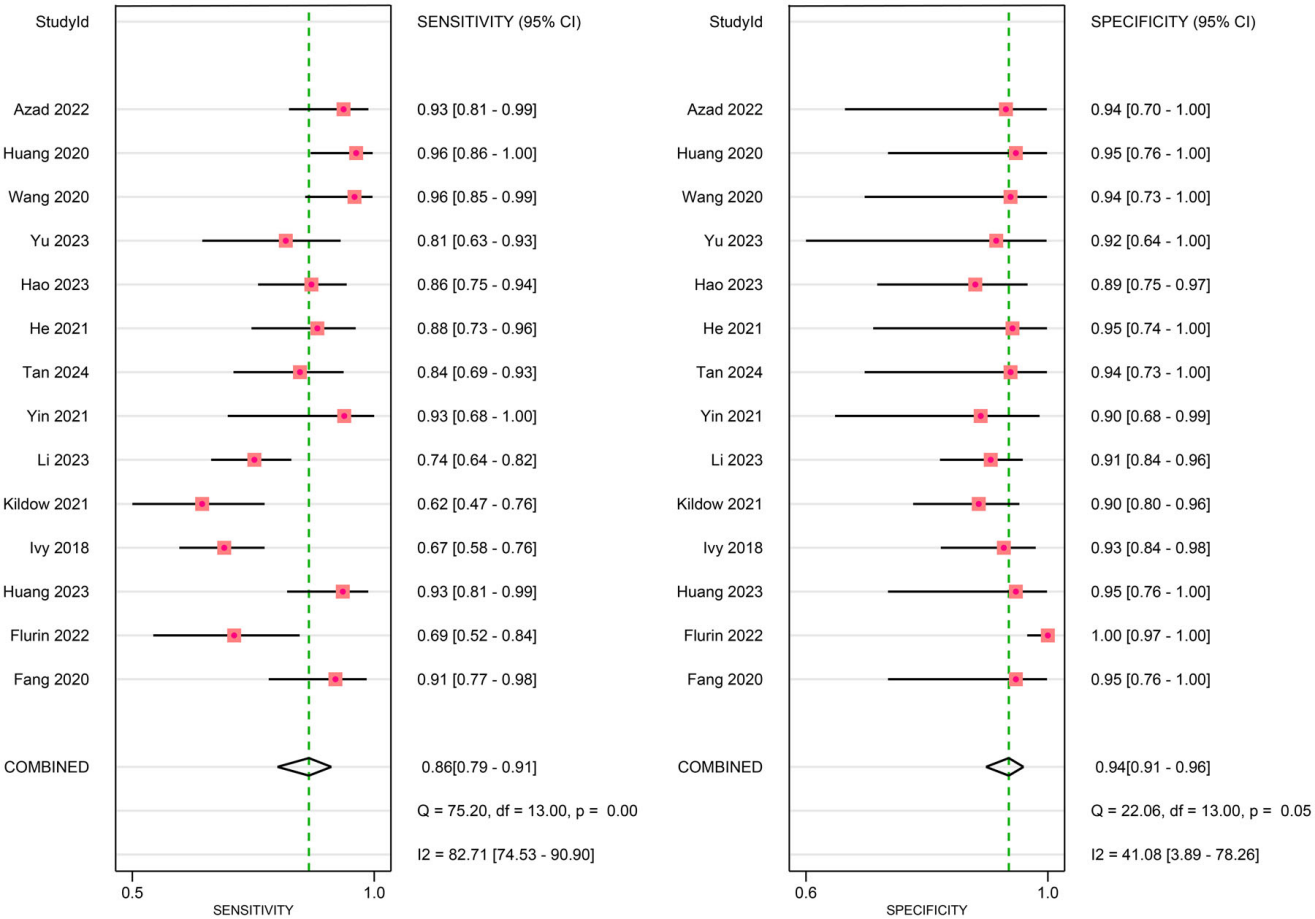
Forest plots of sensitivity and specificity of synovial fluid sample. CI, confidence interval.

**Figure 4:**
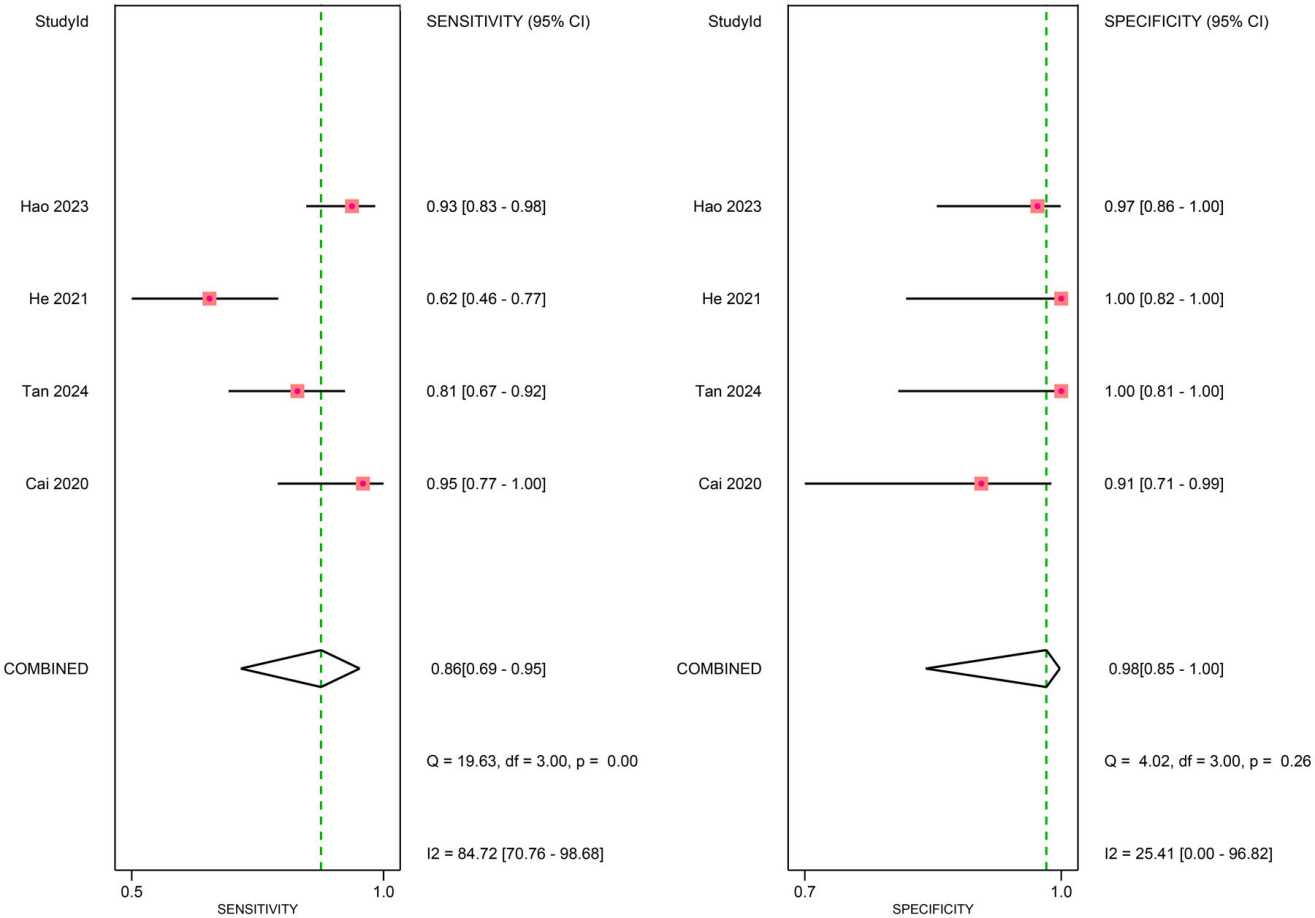
Forest plots of sensitivity and specificity of periprosthetic tissue sample. CI, confidence interval.

**Figure 5:**
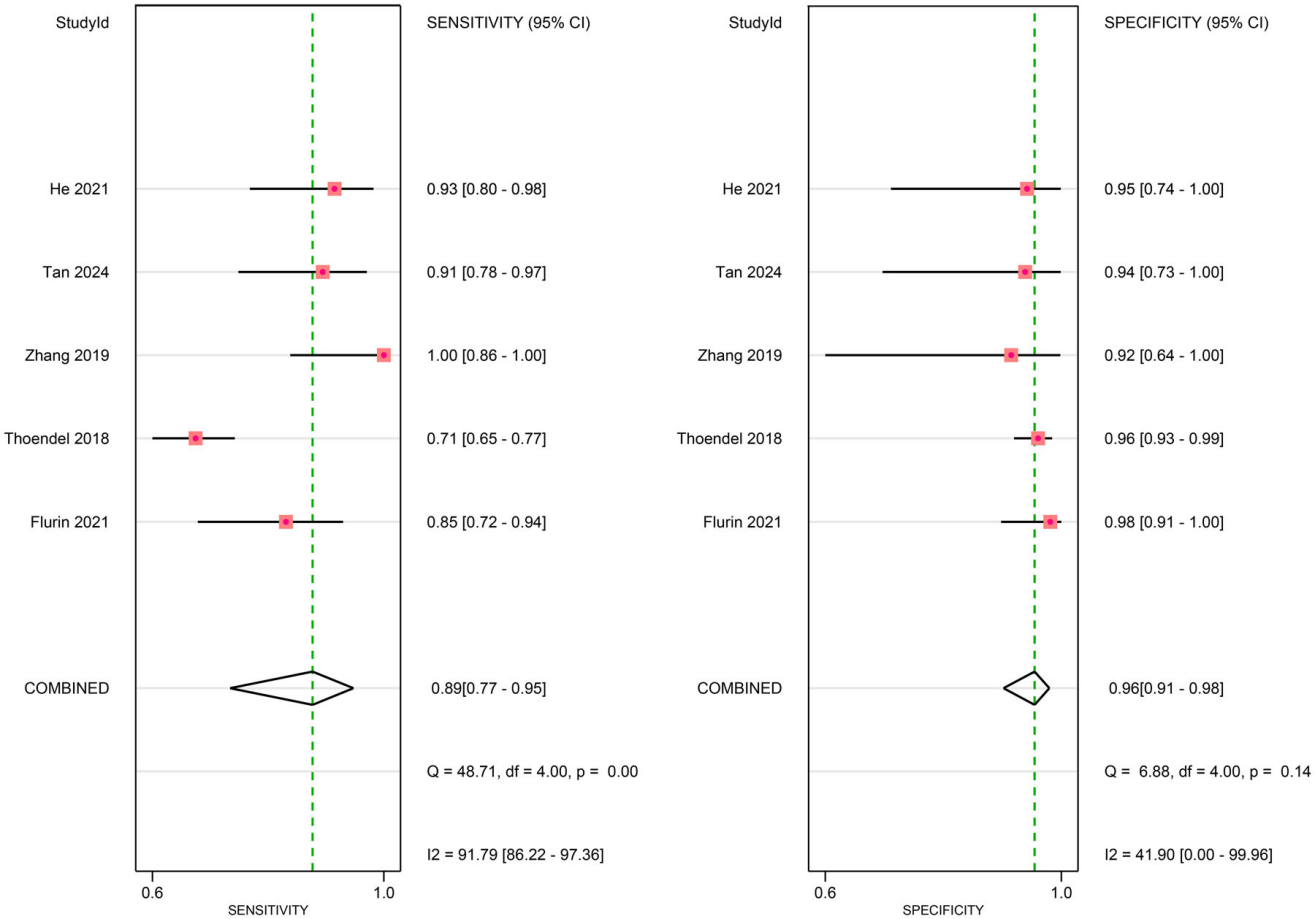
Forest plots of sensitivity and specificity of sonicate fluid sample. CI, confidence interval.

**Figure 6:**
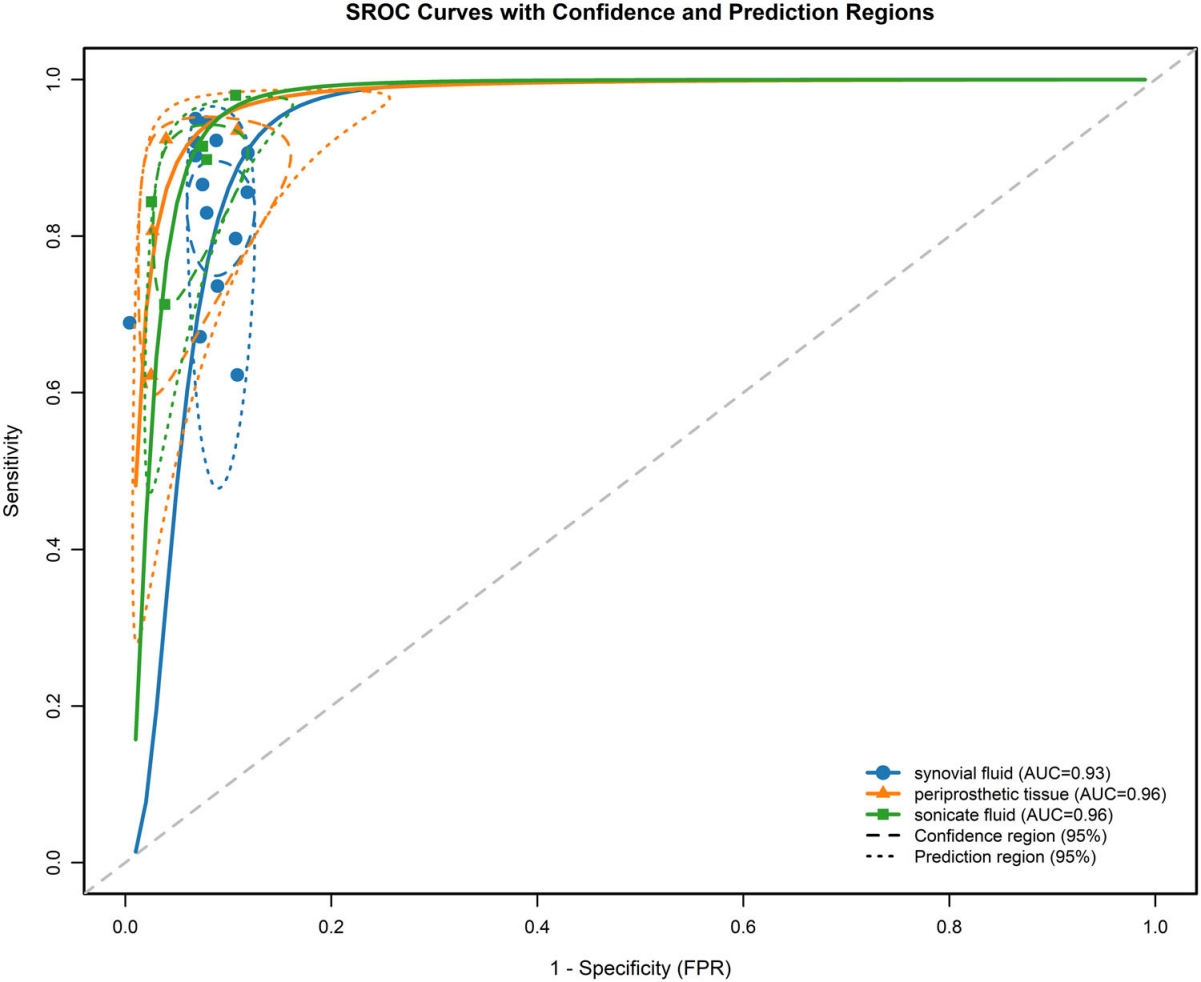
The summary receiver operating characteristic curve of each next‐generation sequencing (NGS) sample for diagnosing periprosthetic joint infection with the corresponding 95% confidence region and 95% prediction region.

**Figure 7:**
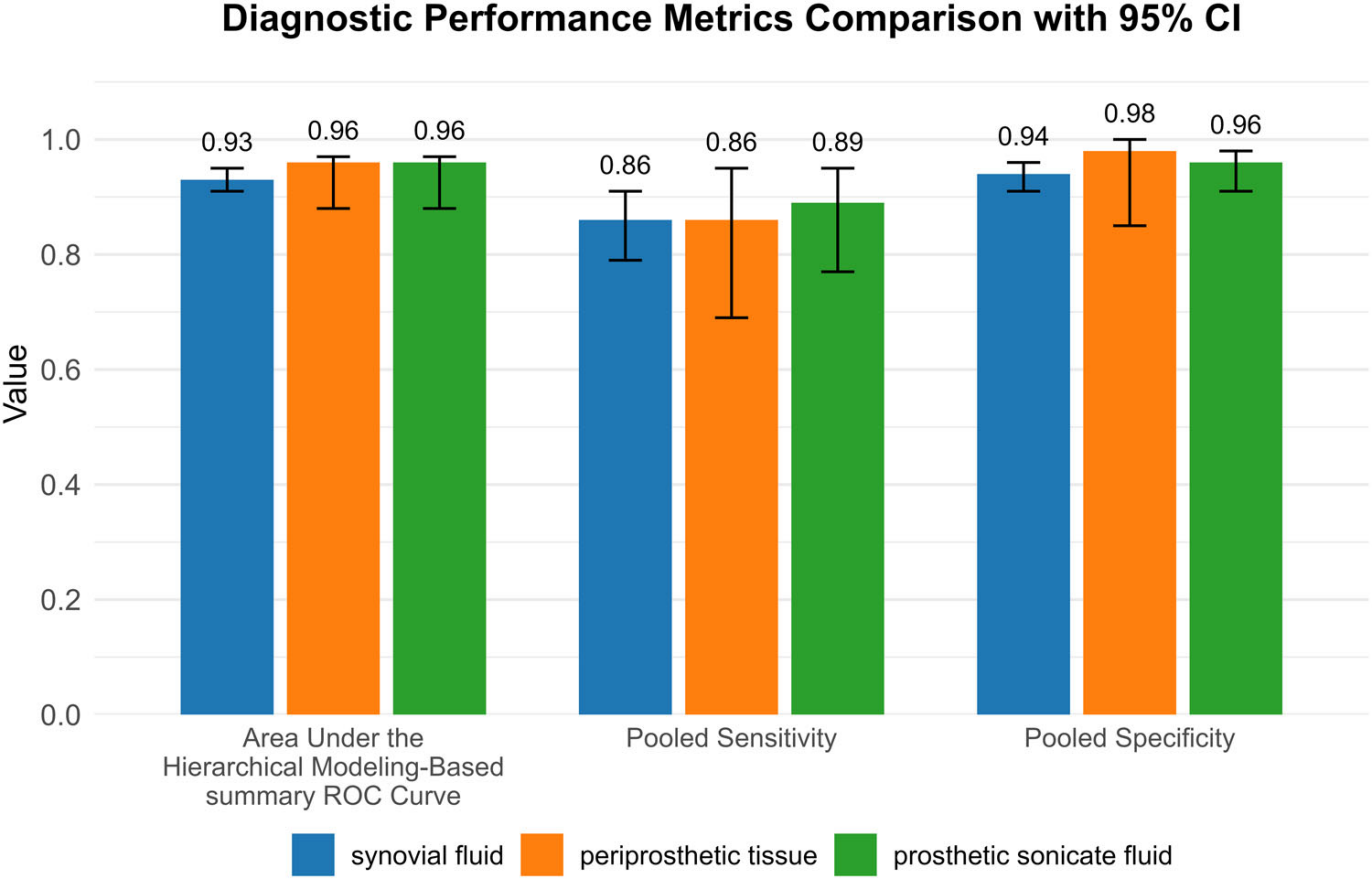
The area under the hierarchical summary receiver‐operating characteristic curve, along with the pooled sensitivity and specificity of next‐generation sequencing for diagnosing periprosthetic joint infection (PJI), are presented. Vertical lines indicate 95% confidence intervals (CIs). ROC, receiver operating characteristic.

Figures 3, 4, 5 present the sensitivity and specificity distributions across studies, with pooled estimates demonstrating: synovial fluid NGS sensitivity 0.86 (95% CI: 0.79–0.91) and specificity 0.94 (95% CI: 0.91–0.96); periprosthetic tissues NGS sensitivity 0.86 (95% CI: 0.69–0.95) and specificity 0.98 (95% CI 0.85–1.00); sonicate fluid NGS sensitivity 0.89 (95% CI: 0.77–0.95) and specificity 0.96 (95% CI: 0.91–0.98). SROC analysis revealed AUC values of 0.93(95% CI: 0.89–0.95), 0.96(95% CI: 0.88–0.97) and 0.96(95% CI: 0.88–0.97) for synovial fluid, periprosthetic tissues and sonicate fluid, respectively (Table 2, and Figures 6 and 7). Utilising the DeLong's test, we found no statistically significant differences in AUC values between synovial fluid, periprosthetic tissue and sonicate fluid (all pairwise comparisons *p* > 0.05: 0.24, 0.78 and 0.37, respectively).

**Table 2:** Meta‐analysis results.

Sample type	Study	Sample	AUC	Sensitivity	Specificity
Synovial fluid	14	1246	0.93 (0.91–0.95)	0.86 (0.79–0.91)	0.94 (0.91–0.96)
Periprosthetic tissues	4	259	0.96 (0.88–0.97)	0.86 (0.69–0.95)	0.98 (0.85–1.00)
Sonicate fluid	5	670	0.96 (0.88–0.97)	0.89 (0.77–0.95)	0.96 (0.91–0.98)

Abbreviation: AUC, area under the hierarchical modelling‐based summary receiver‐operating characteristic curve.

### Sensitivity analysis and subgroup analysis

**Figure 8:**
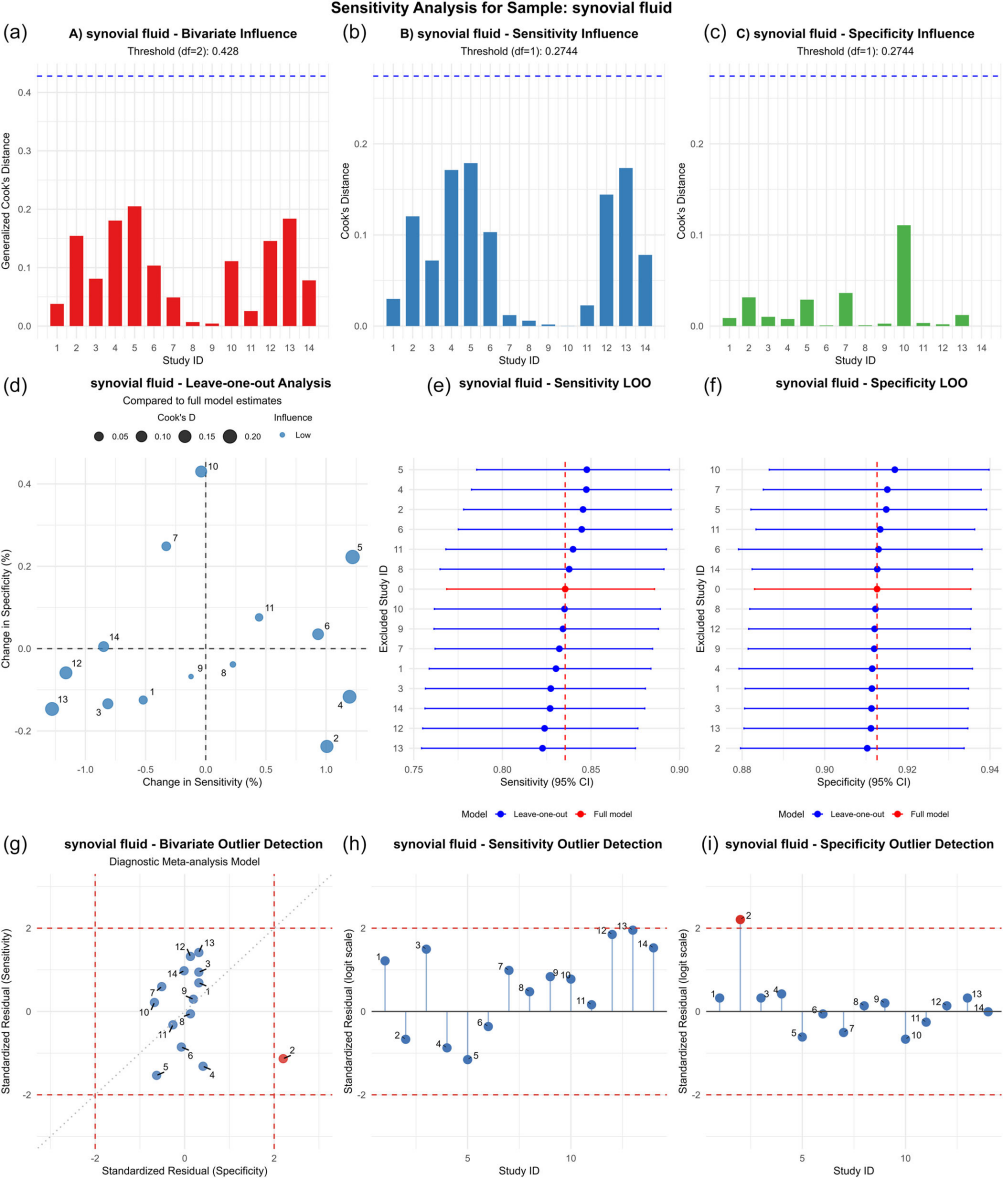
Sensitivity analysis for synovial fluid: diagram of (a–c), influence analysis; (d–f), leave‐one‐out analysis; (g–i), outlier detection for synovial fluid, respectively.

**Figure 9:**
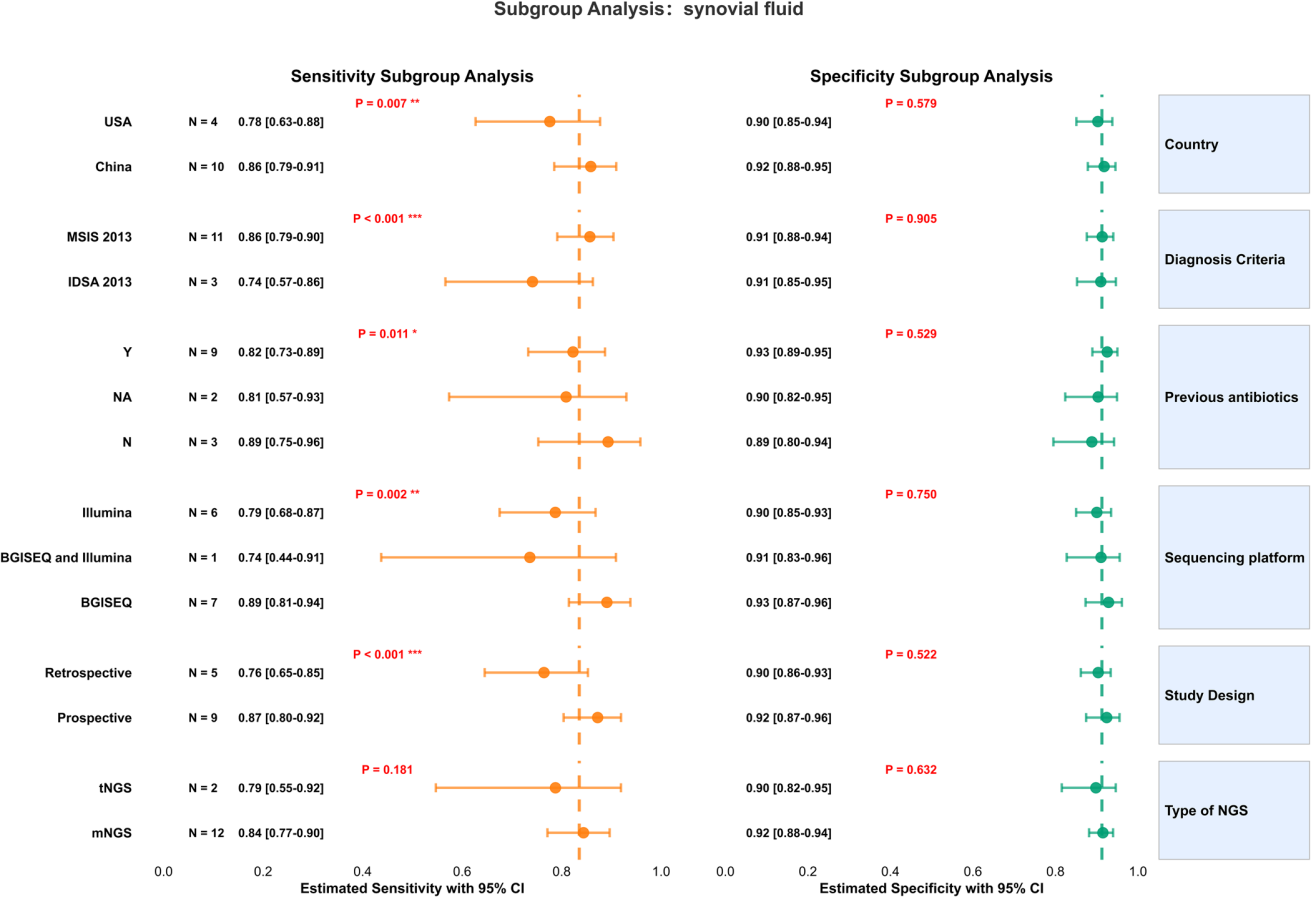
Subgroup analysis of synovial fluid. IDSA, Infectious Diseases Society of America; mNGS, metagenomic next‐generation sequencing; MSIS, Musculoskeletal Infection Society; N, no; NA, not available; tNGS, targeted next‐generation sequencing. Y, yes.

Although significant heterogeneity in sensitivity and specificity was detected between studies, we employed a random‐effects model; thus, the presence of heterogeneity was deemed acceptable. We performed sensitivity and subgroup analyses to investigate heterogeneity. Sensitivity analyses across sample types (Figure 8 and Supporting Information: Figures S2 and S3) identified the study by Flurin et al. [12] as a potential heterogeneity source for synovial fluid. Subsequent subgroup analyses (with low heterogeneity defined as *p* > 0.05; detailed in Figure 9 and Supporting Information: Figures S4 and S5) revealed significant associations: sensitivity heterogeneity for synovial fluid correlated with study design, diagnostic criteria, country, prior antibiotic exposure, and sequencing platform (*p* < 0.05); sonicate fluid heterogeneity was influenced by study design, country, diagnostic criteria, and platform (*p *< 0.05); while periprosthetic tissue heterogeneity was primarily affected by antibiotic exposure. Key subgroup findings demonstrated: (1) elevated synovial fluid sensitivity in Chinese prospective studies using MSIS criteria, antibiotic‐excluded cohorts, and BGISEQ platforms (*p* < 0.05); (2) higher sonicate fluid sensitivity in Chinese prospective studies applying MSIS criteria with BGISEQ (*p* < 0.05); and (3) improved periprosthetic tissue sensitivity following antibiotic exclusion (*p* < 0.05).

### Publication bias

Funnel plot analysis demonstrated no significant publication bias across specimen types (synovial fluid: *p* = 0.07; periprosthetic tissues: *p* = 0.28; sonicate fluid: *p* = 0.06; see Supporting Information: Figure S6A–C).

## DISCUSSION

This meta‐analysis synthesises the diagnostic performance of NGS applied to different specimen types for PJI. Numerically, sonicate fluid yielded the highest pooled sensitivity (0.89, 95% CI: 0.77–0.95), whereas periprosthetic tissue showed the highest specificity (0.98, 95% CI: 0.85–1.00); All three specimen types achieved high diagnostic accuracy (pooled sensitivity ≥ 0.86, specificity ≥ 0.94, AUC ≥ 0.93), pairwise comparisons of AUCs using DeLong's test revealed no statistically significant differences (*p* > 0.05), supporting the clinical utility of NGS in PJI diagnosis. The choice among specimen types necessitates consideration of the sensitivity‐specificity trade‐off in the clinical context.

This meta‐analysis establishes that NGS of sonicate fluid achieves numerically higher pooled sensitivity (0.89, 95% CI: 0.77–0.95) than synovial fluid or periprosthetic tissue NGS, while maintaining strong specificity (0.96, 95% CI: 0.91–0.98) and an excellent AUC (0.96, 95% CI: 0.88–0.97). This supports its value for infection detection and aligns with studies demonstrating enhanced pathogen detection through sonication‐mediated biofilm disruption [17, 30, 43, 54], primarily due to sonication‐driven biofilm disruption enhancing microbial DNA yield. Critically, the microbial identification provided by NGS carries implications for perioperative management, as detection of specific pathogens directly informs optimal antibiotic selection. While this approach increases microbial biomass for sequencing and improves sensitivity, critical limitations must be emphasised: (1) Specimen acquisition necessitates implant removal, precluding preoperative use in DAIR procedures or initial therapy planning; (2) false positive risks from environmental contaminants, human DNA interference [16], or detection of non‐viable organisms—independent of test sensitivity—potentially causing misdiagnosis and over‐treatment; (3) the clinical significance of NGS‐positive results remains unvalidated without correlation with long‐term outcomes such as infection recurrence rates. Therefore, although sonicate fluid shows analytical promise, its routine clinical application necessitates rigorous contamination controls and requires validation of false positive rates and therapeutic impact, and results must be interpreted within comprehensive clinical contexts rather than as standalone determinants.

Periprosthetic tissues—routinely collected during revision arthroplasty [36]—demonstrated the highest pooled specificity (0.98, 95% CI: 0.85–1.00) in our analysis, making a positive result highly reliable for confirming infection. They also showed clinically acceptable sensitivity (0.86, 95% CI: 0.69–0.95) and an excellent AUC (0.96, 95% CI: 0.88–0.97). While Cai et al. [3] reported diagnostic advantages of multiple deep‐tissue specimens over synovial fluid, He et al. [17] observed that periprosthetic tissue exhibited the lowest sensitivity among synovial fluid, tissue, and sonicate fluid when tested by mNGS. This apparent discrepancy arises primarily from methodological variations, including (1) sampling heterogeneity: diagnostic yield varies significantly with collection site, with inflamed areas (e.g., those showing 18F‐fluorodeoxy glucose positron emission tomography/computed tomography uptake [5]) yielding higher accuracy. (2) Host DNA interference: compared to synovial fluid, tissue samples contain a higher proportion of human DNA, which can dilute microbial reads and potentially reduce mNGS sensitivity [16]. Consequently, homogenisation of tissues and selective degradation of host DNA prior to extraction may improve mNGS detection rates [16]. Crucially, despite the inter‐study sensitivity variability observed in individual reports, our meta‐analysis demonstrates that periprosthetic tissue mNGS can achieve high sensitivity (0.86; 95% CI: 0.69–0.95) and exceptional specificity (0.98; 95% CI: 0.85–1.00). This positions it as a pivotal diagnostic tool: A negative result can help rule out infection, but requires cautious interpretation given the potential for FN; conversely, a positive result significantly increases diagnostic certainty (especially when concordant with other findings) and effectively rules in infection.

Current diagnostic criteria for PJI—including those from the MSIS [35], European Bone and Joint Infection Society (EBJIS) [31], ICM [34], IDSA [33] and European Society of Clinical Microbiology and Infectious Diseases—predominantly rely on intraoperative findings (tissue biopsy, implant sonication, purulence and histopathology) [39]. Yet preoperative pathogen identification remains essential for optimal surgical planning and antibiotic stewardship. Our analysis demonstrates synovial fluid NGS provides high specificity (0.94, 95% CI: 0.91–0.96) but suboptimal sensitivity (0.86, 95% CI: 0.79–0.91), implying that whereas a positive result may strongly support PJI, a negative result cannot exclude infection due to FN risks from low microbial biomass or prior antibiotic exposure. Consequently, synovial fluid NGS alone is insufficient for screening, and treatment decisions based solely on negative findings are clinically unjustified. Nevertheless, synovial fluid remains the only preoperative sample enabling organism identification to guide perioperative antibiotics. Though biomarkers like calprotectin (sensitivity 98.1%, specificity 95.7%) [46] and alpha‐defensin (sensitivity 96%, specificity 95%) [53] show excellent diagnostic performance, NGS provides the critical advantage of microbial identification—enabling targeted antimicrobial therapy unattainable through biomarker testing.

Consistent with previous studies reporting that NGS of sonicate fluid detects more pathogenic microorganisms than NGS of synovial fluid and periprosthetic tissue [23, 43], our meta‐analysis confirms distinct diagnostic profiles across specimen types for PJI diagnosis: sonicate fluid NGS demonstrates higher sensitivity (0.89 vs. synovial fluid/tissue: 0.86)—aligning with enhanced pathogen detection through biofilm liberation—though this requires weighing against false positive risks from environmental contaminants; while periprosthetic tissue NGS achieves exceptional specificity (0.98) with acceptable sensitivity critical for minimising false‐positive diagnoses. Critically, diagnostic yield is maximised through combined intraoperative analysis of sonicate fluid and multiple periprosthetic tissues (≥ 3 sites [3]), where sonicate fluid broadens pathogen detection and multi‐site tissue sampling enhances specificity via concordance validation, thereby addressing sampling heterogeneity and mitigating false results. Consequently, we propose: during explantation, collect sonicate fluid and multiple deep‐tissue specimens for parallel NGS; preoperatively, utilise synovial fluid NGS when feasible (acknowledging screening limitations, sensitivity 0.86); and optimise workflows with host DNA depletion for tissues [16] and sterile processing for sonicate fluid.

According to the different characteristics of the study, we performed subgroup analyses stratified by major categories. For synovial fluid and sonicate fluid samples, sensitivity was significantly higher in studies conducted in China than in those from the United States. Significant differences were also observed across subgroups defined by study design, PJI diagnostic criteria (MSIS and IDSA), and sequencing platform. In analyses of synovial and periprosthetic tissues, prior antibiotic use was also associated with significant performance differences. Notably, a majority of US‐based studies employed retrospective designs, predominantly utilised IDSA diagnostic criteria, and relied on Illumina sequencing platforms. We speculate that the observed performance differences may be attributable, at least in part, to variations in sequencing platforms, as different technologies exhibit distinct detection efficiencies. Furthermore, subgroup analysis comparing the two primary NGS methodologies revealed no statistically significant difference between them (*p *> 0.05). While several meta‐analyses evaluating the diagnostic value of NGS have been published [14, 29, 41], these studies are limited by small sample sizes, inconsistent PJI diagnostic criteria, and poorly defined NGS methodologies. Critically, they also fail to adequately classify sample types and conduct the necessary in‐depth investigations required to draw more specific and compelling conclusions.

Our study has potential limitations. The overall quality of the literature and the risk of bias included in this study were relatively acceptable, but potential methodological biases cannot be excluded. The small number of eligible studies reduces the generalisability of our findings. However, we have included all available studies known to date performed with NGS for PJI. The presence of heterogeneity in sensitivity and specificity across the included studies may affect the diagnostic accuracy and lead to bias. However, our application of bivariate random‐effects models appropriately accounted for this variability. Methodological heterogeneity predominantly arose from variations in NGS techniques, inconsistent reporting of preoperative antibiotic use, differences in study design and geographic regions, and PJI diagnostic criteria (MSIS/IDSA). Although sensitivity and subgroup analyses were performed, significant residual heterogeneity persisted, potentially thereby introducing bias. Despite these constraints, our conclusions align with existing literature regarding the diagnostic utility of synovial fluid, periprosthetic tissues and sonicate fluid in PJI diagnosis. This consistency across methodological differences strengthens the credibility of our specimen‐stratified accuracy estimates.

## CONCLUSION

This meta‐analysis reveals complementary roles of specimen types for NGS in PJI diagnosis: Sonicate fluid demonstrates higher sensitivity compared with periprosthetic tissue and preoperative fluid, while maintaining strong specificity, making it valuable for detecting infection. Periprosthetic tissue provides exceptional specificity with acceptable sensitivity, confirming its value in infection confirmation. All specimens show clinically useful AUC values. Sonicate fluid shows promise, but specimen selection warrants careful consideration of the sensitivity‐specificity trade‐off in clinical practice and requires validation of clinical utility due to the absence of a perfect PJI diagnostic gold standard and the risk of false positives. Understanding these sample‐specific diagnostic properties is crucial for optimising microbiological assessment and preventing misdiagnosis. Future standardised studies correlating NGS results with treatment outcomes are needed to define clinical value.

## AUTHOR CONTRIBUTIONS

Lina Wang and Kun Song conceived the original ideas of this manuscript. Shangxiang Feng and Zhongyuan Zhao screened out eligible studies separately. Lina Wang, Kun Song, Shangxiang Feng, Shengjie Dong and Yuchi Zhao discussed the controversial parts of literature screening and quality evaluation. Kun Song, Shangxiang Feng, Shengjie Dong and Yuchi Zhao completed data collection, analysis and finished the manuscript. Lina Wang and Zhongyuan Zhao supervised the entire process and revised the manuscript. All authors have read and approved the manuscript.

## CONFLICT OF INTEREST STATEMENT

The authors declare no conflict of interest.

## ETHICS STATEMENT

This article does not contain any studies with human participants or animals performed by any of the authors.

## Supporting information

Figure S1 A.

Figure S1 B.

Figure S1 C.

Figure S2.

Figure S2.

Figure S3.

Figure S3.

Figure S4.

Revised Supplementary Material.

## Data Availability

The data sets used and/or analysed during the current study are available from the corresponding author upon reasonable request.
