# Supplementary figures and images for: Comparative diagnostic accuracy of next‐generation sequencing in different specimen types for periprosthetic joint infection: A systematic review and meta‐analysis
Source: Knee Surg Sports Traumatol Arthrosc. 2025 Oct 17;34(5):1725–39. doi: 10.1002/ksa.70095 (PMC13122749; doi:10.1002/ksa.70095)

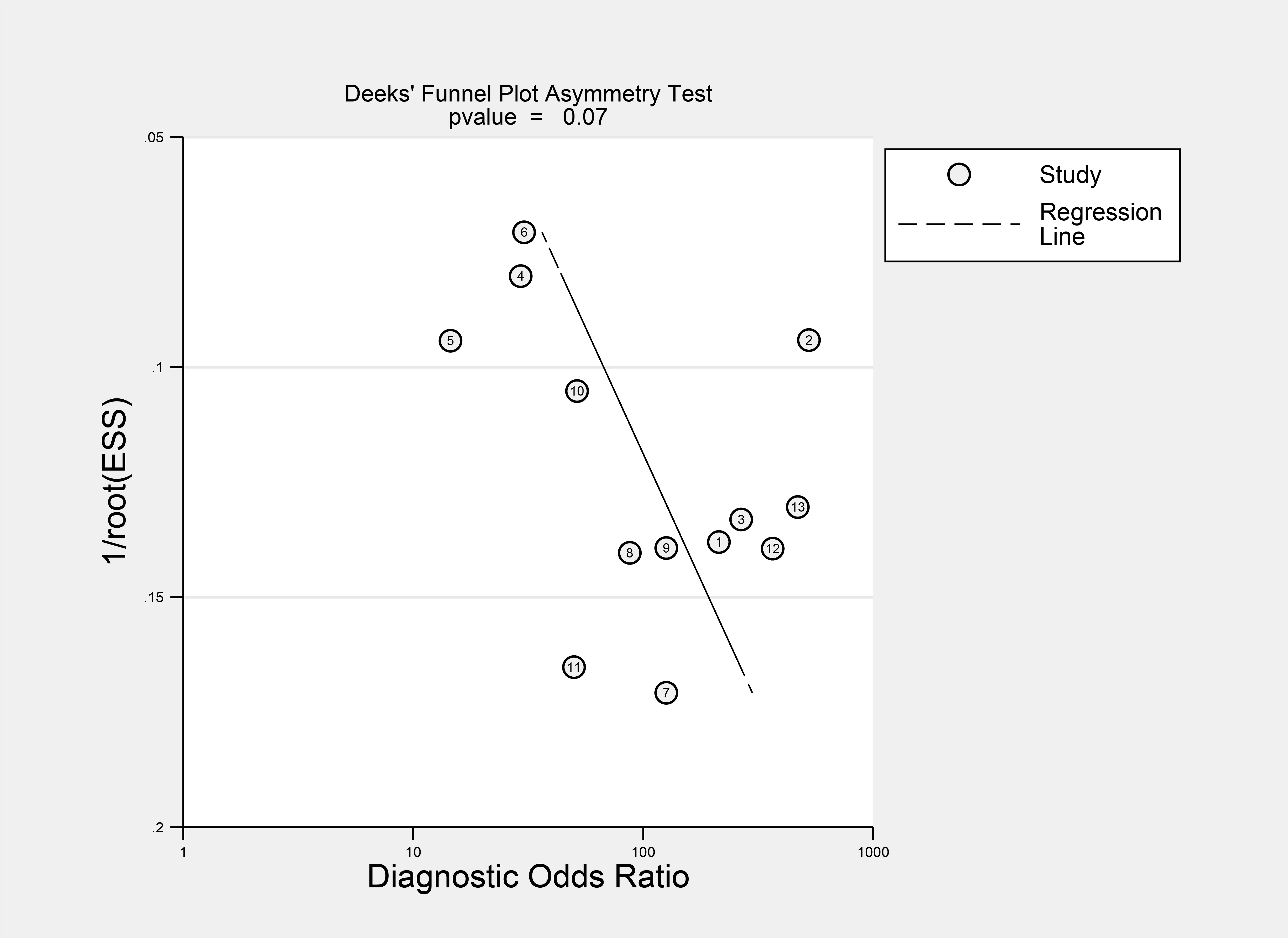

Supplement: Supplementary file 1 — Figure S1 A. [file KSA-34-1725-s001.tif]

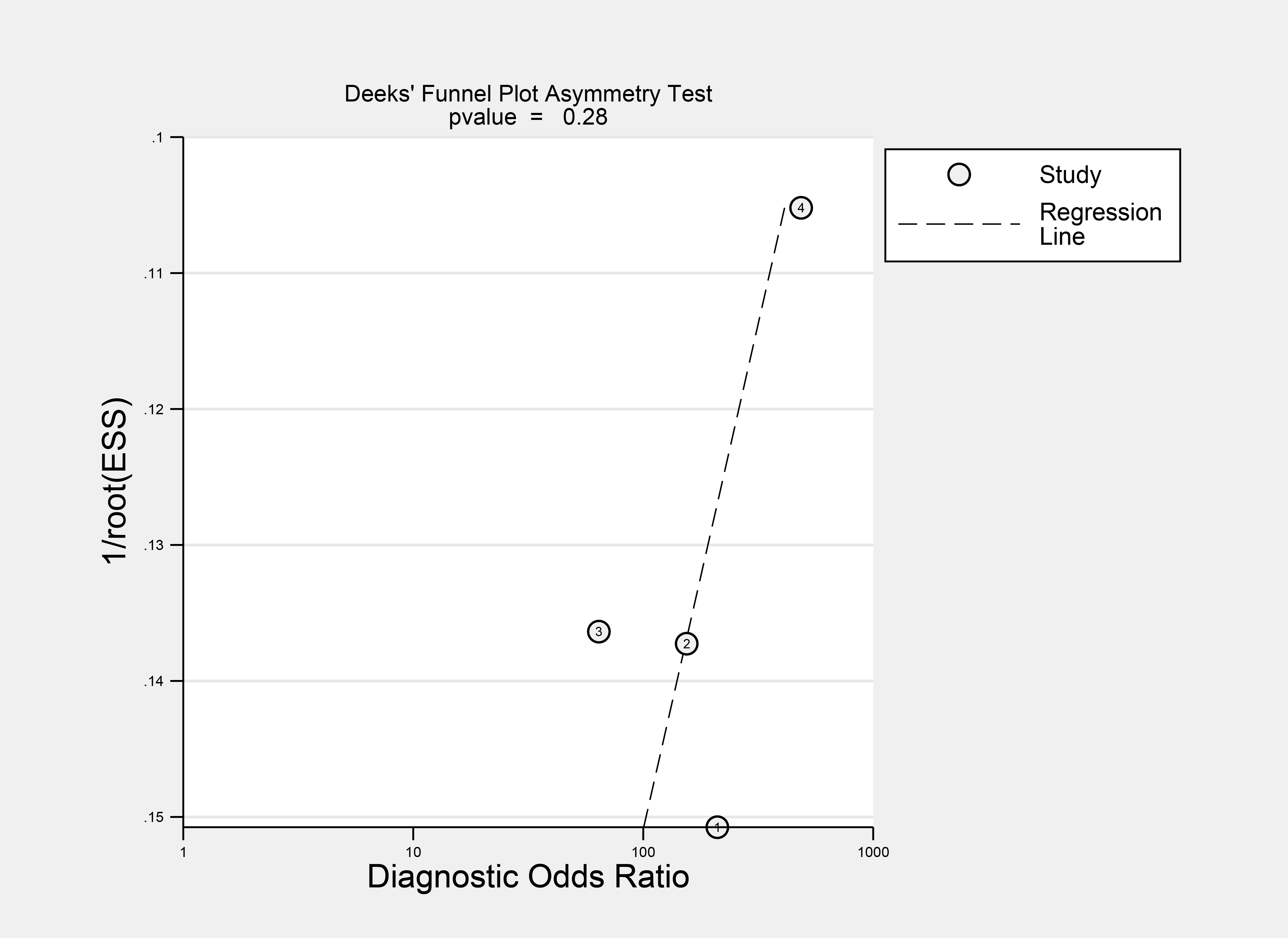

Supplement: Supplementary file 2 — Figure S1 B. [file KSA-34-1725-s003.tif]

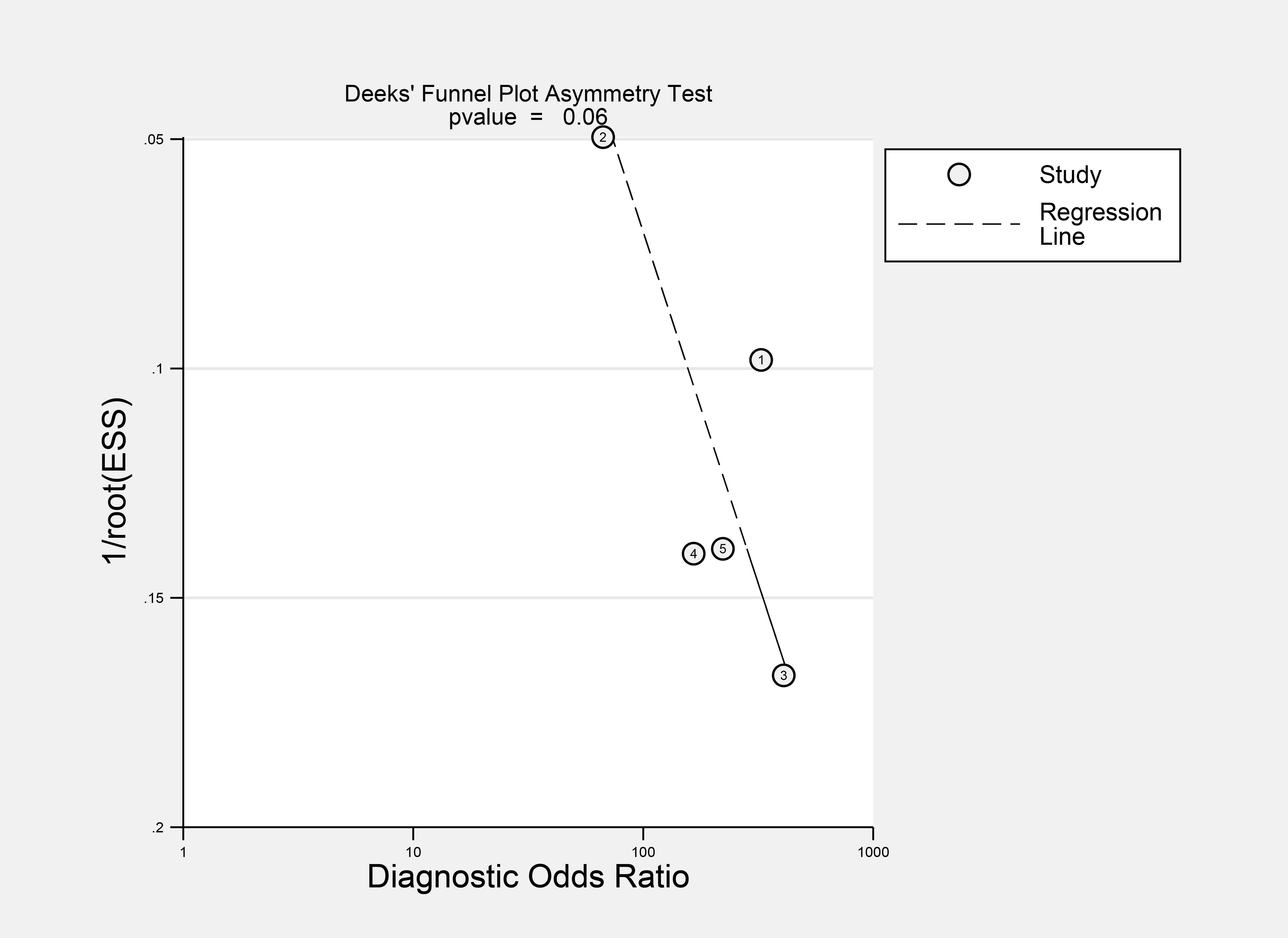

Supplement: Supplementary file 3 — Figure S1 C. [file KSA-34-1725-s007.tif]
