# Supplementary material for: Comparative diagnostic accuracy of next‐generation sequencing in different specimen types for periprosthetic joint infection: A systematic review and meta‐analysis
Source: Knee Surg Sports Traumatol Arthrosc. 2025 Oct 17;34(5):1725–39. doi: 10.1002/ksa.70095 (PMC13122749; doi:10.1002/ksa.70095)
Supplement: Supplementary file 9 — Revised Supplementary Material. [file KSA-34-1725-s004.docx]

**Supplement materials**

**Comparative Diagnostic Accuracy of Next-Generation Sequencing in Different Specimen Types for Periprosthetic Joint Infection: A Systematic Review and Meta-Analysis**

Contents

[Supplement materials 1](#_Toc18136)

[Search strategies 2](#_Toc3869)

[PJI criteria 3](#_Toc19430)

[Figure S1 5](#_Toc10861)

[Figure S2 6](#_Toc3873)

[Figure S3 7](#_Toc25440)

[Figure S4 8](#_Toc5633)

[Figure S5 9](#_Toc3249)

[Figure S6 9](#_Toc26332)

[Table 1 11](#_Toc8154)

[Table 2 12](#_Toc22481)

[Table 3 13](#_Toc6855)

**Search strategies**

**Filter: search period: inception – 2025/6/1**

**Searching in PubMed, MEDLINE:**

((prosthesis-related infection*[Mesh]) OR (Periprosthetic Joint Infection*[Title/Abstract]) OR (Prosthetic Joint Infection*[Title/Abstract]) OR (PJI[Title/Abstract]) OR (prosthesis infection*[Title/Abstract]) OR (prosthesis related infection*[Title/Abstract]) OR (periprosthetic infection*[Title/Abstract])OR (septic loosening[Title/Abstract]) OR(joint infection[Title/Abstract]))

**AND**

(("High-Throughput Nucleotide Sequencing"[Mesh])OR(next-generation sequencing[Title/Abstract]) OR (Generation Sequencing[Title/Abstract]) OR (NGS[Title/Abstract]) OR (metagenomic sequencing[Title/Abstract]) OR (metagenomic next-generation sequencing[Title/Abstract]) OR (mNGS[Title/Abstract]) OR (shotgun metagenomics[Title/Abstract]) OR (tNGS[Title/Abstract]) OR (sequencing[Title/Abstract]))

**Searching in Embase, EMTREE:**

('periprosthetic joint infection'/exp OR ‘Periprosthetic joint infection’:ab,ti,kw OR ‘Prosthetic joint infection*’:ab,ti,kw OR 'PJI’:ab,ti,kw OR ‘prosthesis infection*’:ab,ti,kw OR ‘prosthesis related infection*’:ab,ti,kw OR ‘periprosthetic infection*’:ab,ti,kw OR ‘septic loosening’:ab,ti,kw OR ‘joint infection’:ab,ti,kw)

**AND**

('high throughput sequencing'/exp OR ‘next-generation sequencing’:ab,ti,kw OR ‘Generation Sequencing’:ab,ti,kw OR ‘NGS’:ab,ti,kw OR ‘metagenomic sequencing’:ab,ti,kw OR ‘metagenomic next-generation sequencing’:ab,ti,kw OR ‘mNGS’:ab,ti,kw OR ‘shotgun metagenomics’:ab,ti,kw OR ‘tNGS’:ab,ti,kw OR ‘sequencing’:ab,ti,kw)

**Searching in Cochrane Central Register of Controlled Trials (CENTRAL)**

((MeSH descriptor: [Prosthesis-Related Infections] explode all trees) OR ((Periprosthetic Joint Infection* OR Prosthetic Joint Infection* OR PJI OR prosthesis infection* OR prosthesis related infection* OR periprosthetic infection* OR septic loosening OR joint infection):ti,ab,kw))

**AND**

((MeSH descriptor: [High-Throughput Nucleotide Sequencing] explode all trees) OR (next-generation sequencing):ti,ab,kw OR (Generation Sequencing):ti,ab,kw OR (NGS):ti,ab,kw OR (metagenomic sequencing):ti,ab,kw OR (metagenomic next-generation sequencing):ti,ab,kw OR (mNGS):ti,ab,kw OR (shotgun metagenomics):ti,ab,kw OR (tNGS):ti,ab,kw OR (sequencing):ti,ab,kw

**PJI criteria**

**European Bone and Joint Infection Society (EBJIS) Criteria(1)**

| **Parameter** | **Infection Unlikely (all findings negative)** | **Infection Likely 2 findings positive)** | **Infection Confirmed any positive finding)** |
| --- | --- | --- | --- |
| **Clinical and Blood Workup** | **Clinical features** • Clear alternative reason for implant dysfunction (eg, fracture, implant breakage, malposition, tumor) | **Clinical features** • Radiologic signs of loosening within the first 5 years  after implantation • Previous wound healing problems • History of recent fever or bacteremia | **Clinical features** • Purulence around the prosthesis • Sinus tract with evidence of communication  to the joint or visualization of the prosthesis |
| **C-reactive protein (CRP)** |  | >10 mg/L (1 mg/dL) | (Not specifically stated for “Confirmed”) |
| **Synovial Fluid Cytological Analysis** | • Leukocyte count ≤1500 cells/µL • PMN ≤65% | • Leukocyte count >1500 cells/µL • PMN >65% | • Leukocyte count >3000 cells/µL • PMN >80% |
| **Synovial Fluid Biomarkers** |  |  | Positive alpha-defensin (immunoassay or lateral-flow assay) |
| **Microbiology** | • Aspiration fluid: No growth • Intraoperative (fluid and tissue): All cultures negative • Sonication: No growth | • Aspiration fluid: Positive culture • Intraoperative (fluid and tissue): Single positive culture • Sonication: >1 CFU/mL of any organism | • Intraoperative (fluid and tissue): ≥2 positive samples with the same organism • Sonication: >50 CFU/mL of any organism |
| **Histology** | High-power field (400×): Negative | High-power field (400×): ≥5 neutrophils in a single high-power field | • High-power field (400×): ≥5 neutrophils in ≥5 high-power fields • Presence of visible microorganisms |
| **Others (Nuclear Imaging)** | Negative 3-phase isotope bone scan | Positive WBC scintigraphy |  |

**Musculoskeletal Infection Society (MSIS) Criteria(2)**

| **Criteria** | **Definition** |
| --- | --- |
| **Overall Definition** | PJI is present if **1 major** criterion is met OR **4 of 6 minor** criteria are met. |
| **Major Criteria** | 1. Two positive periprosthetic cultures with phenotypically identical organisms 2. A sinus tract communicating with the joint |
| **Minor Criteria** | 1. Elevated CRP and ESR 2. Elevated synovial fluid WBC count or “++” change on leukocyte esterase test strip 3. Elevated synovial fluid PMN% 4. Presence of purulence in the affected joint 5. Positive histologic analysis of periprosthetic tissue 6. A single positive culture |

**2013 International Consensus Meeting (ICM) Criteria(3)**

| **Criteria** | **Definition** |
| --- | --- |
| **Overall Definition** | PJI is present if **1 major** criterion is met OR **3 of 5 minor** criteria are met. |
| **Major Criteria** | 1. Two positive periprosthetic cultures with phenotypically identical organisms 2. A sinus tract communicating with the joint |
| **Minor Criteria** | 1. Elevated CRP and ESR 2. Elevated synovial fluid WBC count or “++” change on leukocyte esterase test strip 3. Elevated synovial fluid PMN% 4. Positive histologic analysis of periprosthetic tissue 5. A single positive culture |

**Infectious Diseases Society of America (IDSA) Guidelines(4)**

| **Criteria** |
| --- |
| PJI is present if **1** of the following is met: |
| 1. Sinus tract communicating with the prosthesis |
| 2. Presence of purulence |
| 3. Acute inflammation on histopathologic evaluation of periprosthetic tissue |
| 4. Two or more positive cultures with the same organism (intraoperatively and/or preoperatively) |
| 5. Single positive culture with a virulent organism |

**2018 International Consensus Meeting (ICM) Criteria(5)**

| **Criteria** | **Score** | **Decision** |
| --- | --- | --- |
| **Major Criteria (at least one of the following)** | NA | **Infected** |
| Two positive cultures of the same organism |  |  |
| Sinus tract with evidence of communication to the joint or visualization of the prosthesis |  |  |
| **Preoperative (Minor Criteria)** | (Sum of scores) | **≥6** = Infected  **2–5** = Possibly Infected  **0–1** = Not Infected |
| Elevated CRP or D‐Dimer | 2 |  |
| Elevated ESR | 1 |  |
| Elevated synovial WBC count or LE | 3 |  |
| Positive alpha‐defensin | 3 |  |
| Elevated synovial PMN (%) | 2 |  |
| Elevated synovial CRP | 1 |  |
| **Intraoperative (if inconclusive pre‐op score or dry tap)** | (Sum of scores) | **≥6** = Infected  **4–5** = Inconclusive  **≤3** = Not Infected |
| Positive histology | 3 |  |
| Purulence | 3 |  |
| Single positive culture | 2 |  |

**Figure S1**





**Figure S1** Forest plots of study accuracy results stratified by sample types

**Figure S2**





**Figure S2** Sensitivity Analysis for sonicate fluid: Diagram of (A-C), Influence analysis; (D-F), Leave-one-out Analysis; (G-I), Outlier detection for synovial fluid, respectively

**Figure S3**





**Figure S3** Sensitivity Analysis for periprosthetic tissues: Diagram of (A-C), Influence analysis; (D-F), Leave-one-out Analysis; (G-I), Outlier detection for synovial fluid, respectively

**Figure S4**





**Figure S4** Subgroup analysis of sonicate fluid. IDSA, Infectious Diseases Society of America; MSIS, Musculoskeletal Infection Society; mNGS, metagenomic next-generation sequencing; tNGS, targeted next-generation sequencing.

**Figure S5**





**Figure S5** Subgroup analysis of periprosthetic tissues. Y, yes; N, no;

**Figure S6**

| (A) SNV | 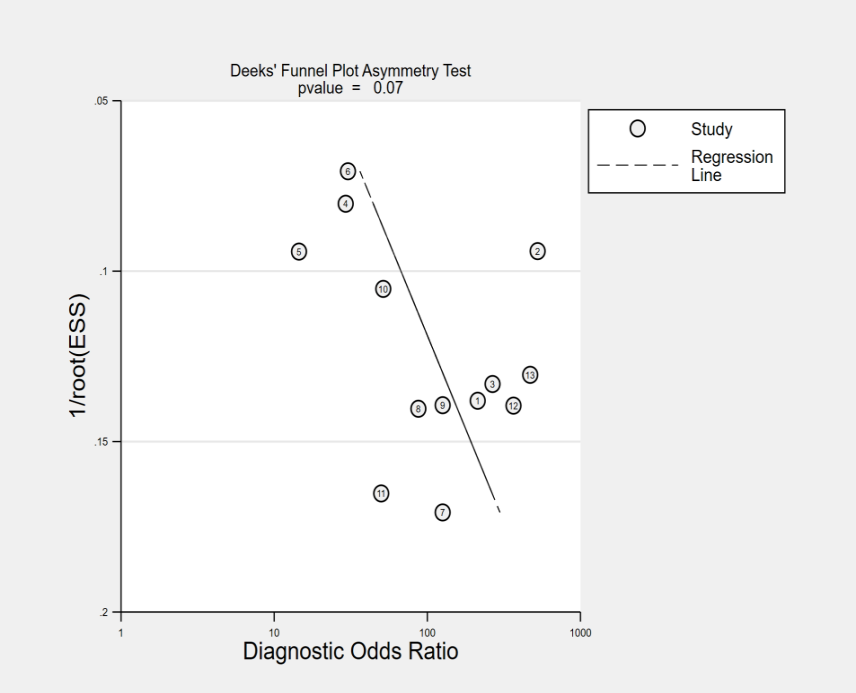 |
| --- | --- |
| (B)  PPT | 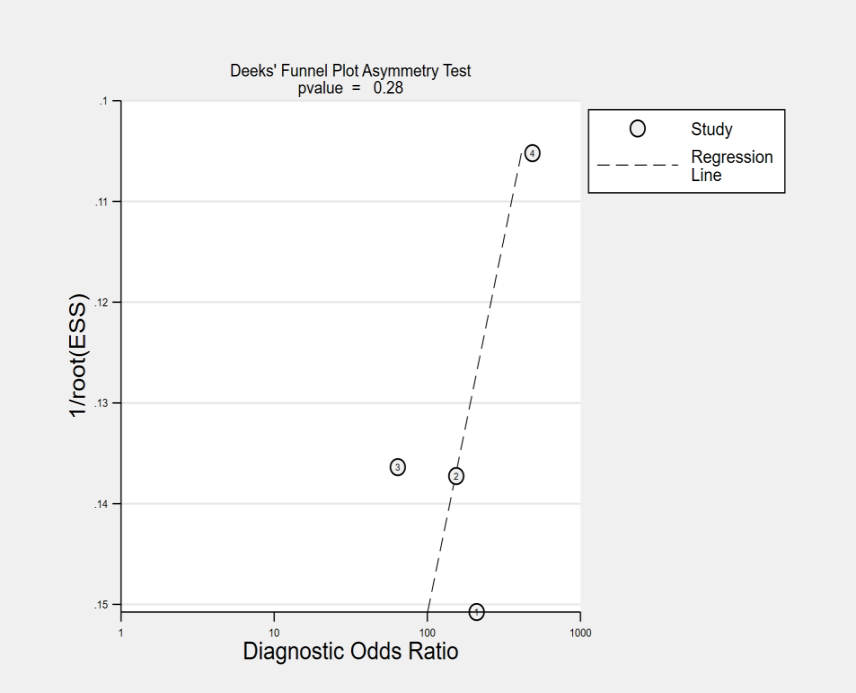 |
| (C) SON | 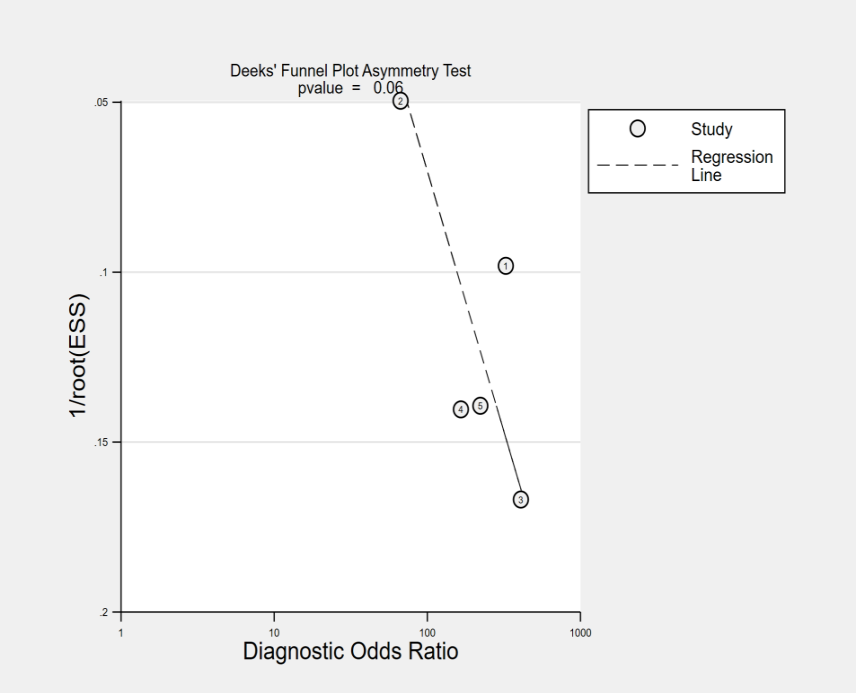 |

**Figure S6** Funnel plots for publication bias. (A) SNV, (B) PPT, (C) SON

Abbreviations: SNV, synovial fluid; PPT, periprosthetic tissues; SON, sonicate fluid.

**Table 1**

**Studies with SNV fluid**

| **Study (Author & Year)** | **Inclusion Interval** | **Study Design** | **Infected/Control joints** | **Diagnosis Criteria** | **Country** | **Sequencing platform** | **Type of NGS** | **Previous antibiotics** | **Sample type** | **Type of Arthroplasty** |
| --- | --- | --- | --- | --- | --- | --- | --- | --- | --- | --- |
| Ivy et al., 2018 | 04/1998 -06/2017 | Retrospective | 107/61 | IDSA 2013 | USA | Illumina | mNGS | Y | SNV fluid | Knee: 168 |
| Wang et al., 2020 | 03/2017-07/2018 | Prospective | 45/18 | MSIS 2013 | China | BGISEQ | mNGS | N | SNV fluid | knee and Hip: NA |
| Fang et al.,2020 | 06/2016-12/2018 | Prospective | 25/13 | MSIS 2013 | China | BGISEQ | mNGS | Y | SNV fluid | Knee: 19 Hip: 19 |
| Huang et al., 2020 | 03/2017-07/2018 | Prospective | 49/21 | MSIS 2013 | China | BGISEQ | mNGS | Y | SNV fluid | Knee: 36 Hip: 34 |
| Kildow et al., 2021 | 01/2017-07/2019 | Retrospective | 48/68 | MSIS 2013 | USA | Illumina | mNGS | N | SNV fluid | Knee: 92 Hip: 24 |
| He et al., 2021 | 10/2017-04/2019 | Prospective | 40/19 | MSIS 2013 | China | BGISEQ | mNGS | Y | SNV fluid | Knee: 34 Hip: 25 |
| Yin et al., 2021 | 07/2017-12/2019 | prospective | 15/20 | MSIS 2013 | China | BGISEQ | mNGS | NA | SNV fluid | Knee: 16 Hip: 19 |
| Flurin et al., 2022 | 08/2020-05/2021 | Retrospective | 36/118 | IDSA 2013 | USA | Illumina | tNGS | Y | SNV fluid | Knee: 96 Hip: 43 shoulder: 14 elbow:1 |
| Azad et al.,2022 | 12/1998-06/2021 | Retrospective | 44/16 | IDSA 2013 | USA | Illumina | tNGS | NA | SNV fluid | Knee: 59 |
| Huang et al., 2023 | 04/2020- 09/2022 | Retrospective | 43/21 | MSIS 2013 | China | BGISEQ and Illumina | mNGS | Y | SNV fluid | Knee: 30 Hip: 34 |
| Li et al., 2023 | 04/2019-04/2021 | Prospective | 107/94 | MSIS 2013 | China | Illumina | mNGS | Y | SNV fluid | Knee: 140 Hip: 61 |
| Yu et al., 2023 | 05/2020-03/2022 | prospective | 31/13 | MSIS 2013 | China | BGISEQ | mNGS | Y | SNV fluid | Knee: 36 Hip: 8 |
| Hao et al., 2023 | 01/2018-01/2021 | Prospective | 58/37 | MSIS 2013 | China | BGISEQ | mNGS | N | SNV fluid | Knee: 56 Hip: 39 |
| Tan et al., 2024 | 09/2021-09/2022 | Prospective | 43/18 | MSIS 2013 | China | Illumina | mNGS | Y | SNV fluid | Knee: 37 Hip: 24 |

**Table 2**

**Studies with PPT**

| **Study (Author & Year)** | **Inclusion Interval** | **Study Design** | **Infected/Control joints** | **Diagnosis Criteria** | **Country** | **Sequencing platform** | **Type of NGS** | **Previous antibiotics** | **Sample type** | **Type of Arthroplasty** |
| --- | --- | --- | --- | --- | --- | --- | --- | --- | --- | --- |
| Cai et al., 2020 | 07/2017-07/2019 | Prospective | 22/22 | MSIS 2013 | China | BGISEQ | mNGS | Y | PPT | Knee: 13 Hip: 31 |
| He et al., 2021 | 10/2017-04/2019 | Prospective | 40/19 | MSIS 2013 | China | BGISEQ | mNGS | Y | PPT | Knee: 34 Hip: 25 |
| Hao et al., 2023 | 01/2018-01/2021 | Prospective | 58/37 | MSIS 2013 | China | BGISEQ | mNGS | N | PPT | Knee: 56 Hip: 39 |
| Tan et al., 2024 | 09/2021-09/2022 | Prospective | 43/18 | MSIS 2013 | China | Illumina | mNGS | Y | PPT | Knee: 37 Hip: 24 |

**Table 3**

**Studies with SON fluid**

| **Study (Author & Year)** | **Inclusion Interval** | **Study Design** | **Infected/Control joints** | **Diagnosis Criteria** | **Country** | **Sequencing platform** | **Type of NGS** | **Previous antibiotics** | **Sample type** | **Type of Arthroplasty** |
| --- | --- | --- | --- | --- | --- | --- | --- | --- | --- | --- |
| Thoendel et al., 2018 | 2011-2016 | Retrospective | 213/195 | IDSA 2013 | USA | Illumina | mNGS | Y | SON fluid | Knee: 281 Hip: 127 |
| Zhang et al., 2019 | 12/2016-12/2018 | prospective | 24/13 | MSIS 2013 | China | BGISEQ | mNGS | Y | SON fluid | Knee: 19 Hip: 318 |
| Flurin et al., 2021 | 05/2007-09/2019 | Retrospective | 47/58 | IDSA 2013 | USA | Illumina | tNGS | Y | SON fluid | elbow: 105 |
| He et al., 2021 | 10/2017-04/2019 | Prospective | 40/19 | MSIS 2013 | China | BGISEQ | mNGS | Y | SON fluid | Knee: 34 Hip: 25 |
| Tan et al., 2024 | 09/2021-09/2022 | Prospective | 43/18 | MSIS 2013 | China | Illumina | mNGS | Y | SON fluid | Knee: 37 Hip: 24 |
